# 2-(3-{(3*R*,4*R*)-4-Methyl-3-[meth­yl(7*H*-pyrrolo­[2,3-*d*]pyrimidin-4-yl)amino]­piperidin-1-yl}oxetan-3-yl)aceto­nitrile monohydrate

**DOI:** 10.1107/S1600536814004449

**Published:** 2014-03-05

**Authors:** Matthias Gehringer, Ellen Pfaffenrot, Peter R. W. E. F. Keck, Dieter Schollmeyer, Stefan A. Laufer

**Affiliations:** a Eberhard-Karls-University Tuebingen, Institute of Pharmacy, Department of Pharmaceutical and Medicinal Chemistry, Auf der Morgenstelle 8, 72076 Tuebingen, Germany; b University Mainz, Institut of Organic Chemistry, Duesbergweg 10-14, 55099 Mainz, Germany

## Abstract

In the title compound, C_18_H_24_N_6_O·H_2_O, the piperidine ring adopts a chair conformation with an N—C—C—C torsion angle of 39.5 (5)° between the *cis*-related substituents. The pyrrole N—H group forms a water-mediated inter­molecular hydrogen bond to one of the N atoms of the annelated pyrimidine ring. The water mol­ecule connects two organic mol­ecules and is disorderd over two positions (occupancies of 0.48 and 0.52). The crystal packing shows zigzag chains of alternating organic and water mol­ecules running parallel to the *a* axis.

## Related literature   

For the biological activity and structure–activity relationships of tofacitinib {systematic name: 3-[(3*R*,4*R*)-4-methyl-3-[meth­yl(7*H*-pyrrolo­[2,3-*d*]pyrimidin-4-yl)amino]­piperidin-1-yl]-3-oxo­propane­nitrile} derivatives, see: Flanagan *et al.* (2010 ▶). For a general overview on the JAK–STAT pathway, see: Shuai & Liu (2003 ▶). The use of oxetanes as carbonyl bioisosteres has been reviewed extensively by Wuitschik *et al.* (2010 ▶). For a recent application of this concept towards tofacitinib-derived JAK3 inhibitors, see: Gehringer *et al.* (2014 ▶).
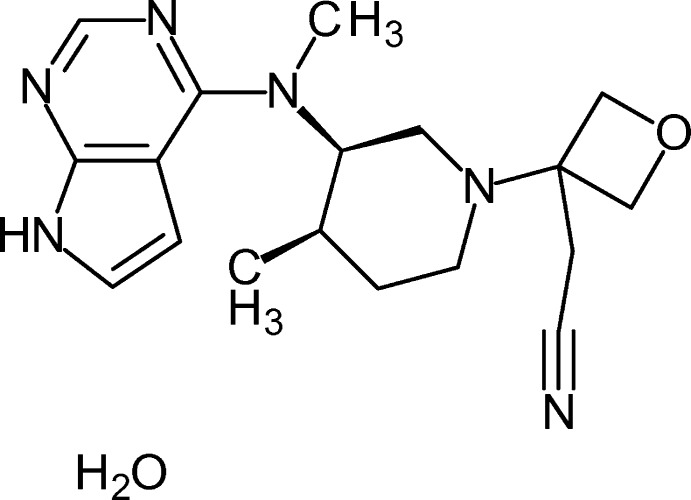


## Experimental   

### 

#### Crystal data  

C_18_H_24_N_6_O·H_2_O
*M* *_r_* = 358.45
Orthorhombic, 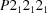

*a* = 6.6088 (6) Å
*b* = 10.1483 (8) Å
*c* = 26.813 (2) Å
*V* = 1798.3 (3) Å^3^
*Z* = 4
Mo *K*α radiation
μ = 0.09 mm^−1^
*T* = 193 K
0.29 × 0.27 × 0.06 mm

#### Data collection  

Stoe IPDS 2T diffractometer
6672 measured reflections
4184 independent reflections
1716 reflections with *I* > 2σ(*I*)
*R*_int_ = 0.079

#### Refinement  

*R*[*F* ^2^ > 2σ(*F* ^2^)] = 0.062
*wR*(*F* ^2^) = 0.150
*S* = 0.90
4184 reflections
246 parameters
H-atom parameters constrained
Δρ_max_ = 0.19 e Å^−3^
Δρ_min_ = −0.22 e Å^−3^

### 

Data collection: *X-AREA* (Stoe & Cie, 2010 ▶); cell refinement: *X-AREA*; data reduction: *X-RED* (Stoe & Cie, 2010 ▶); program(s) used to solve structure: *SIR97* (Altomare *et al.*, 1999 ▶); program(s) used to refine structure: *SHELXL97* (Sheldrick, 2008 ▶); molecular graphics: *PLATON* (Spek, 2009 ▶); software used to prepare material for publication: *PLATON*.

## Supplementary Material

Crystal structure: contains datablock(s) I, Global. DOI: 10.1107/S1600536814004449/bt6965sup1.cif

Structure factors: contains datablock(s) I. DOI: 10.1107/S1600536814004449/bt6965Isup2.hkl

Supporting information file. DOI: 10.1107/S1600536814004449/bt6965Isup3.cml

CCDC reference: 988870

Additional supporting information:  crystallographic information; 3D view; checkCIF report

## Figures and Tables

**Table 1:** Hydrogen-bond geometry (Å, °)

*D*—H⋯*A*	*D*—H	H⋯*A*	*D*⋯*A*	*D*—H⋯*A*
N1—H1⋯O1*L*	0.88	1.90	2.783 (8)	178
N1—H1⋯O2*L*	0.88	2.06	2.816 (7)	144
O1*L*—H1*L*2⋯N8^i^	0.84	2.27	2.868 (8)	129
O2*L*—H2*L*2⋯N8^i^	0.84	2.20	2.733 (7)	121
O2*L*—H2*L*2⋯N25^ii^	0.84	2.43	3.026 (10)	129

Symmetry codes: (i) 
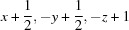
; (ii) 
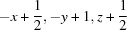
.

**Fig. 1.**
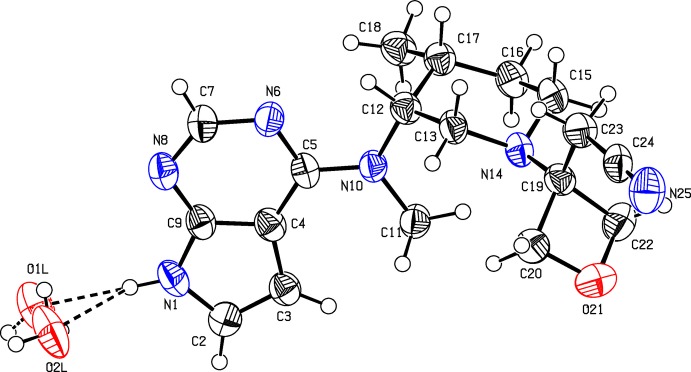
Crystal structure of the title compound with labeling and displacement ellipsoids drawn at the 50% probability level. Water molecules are disorderd with s.o.f. 0.52/0.48.

## supplementary crystallographic information

### 1. Comment

Janus Kinases (JAKs) are non-receptor protein tyrosine kinases mediating signalling through the JAK-STAT (Signal Transducer and Activator of Transcription) pathway. Being crucial signal transducers for a variety of cytokines, growth factors, and interferons, JAKs are involved in numerous pathologies including malignancies, myeloproliferative disorders and autoimmune diseases (Shuai & Liu, 2003). Recently, Tofacitinib (CP690,550; 3-[(3*R*,4*R*)-4-methyl-3-[methyl(7*H*-pyrrolo[2,3-*d*] pyrimidin-4-yl)amino]piperidin-1-yl]-3-oxopropanenitrile) a small-molecule pan-JAK inhibitor was approved by the US Food and Drug Administration for the treatment of rheumatoid athritis (Flanagan *et al.*, 2010). The compound also shows promising results in late stage clinical trials for psoriasis, transplant rejection, and other disorders of the immune system. In search for novel JAK inhibitors, the title compound was prepared as a Tofacitinib bioisostere with altered physicochemical properties (Wuitschik *et al.*, 2010).

In the crystal structure of the title compound, C_18_H_24_N_6_O, the exocyclic amino substituent is oriented almost coplanar to the heteroaromatic ring system with a torsion angle of 0.6 (2)° for C11-N10-C5-C4. The piperidine ring adopts a chair conformation with a torsion angle of 39.5 (5) ° between the *cis* substituents. One of the protons (H23B) of the methylene group between the oxetane ring and the nitrile function is involved in an intermolecular C—H···π interaction while the other methylene proton forms an intermolecular C—H···N interaction with the nitrile group. The oxygen atom of the oxetane ring makes two intermolecular C—H···O contacts with two H atoms (H13A and H15B) of the piperidine ring. The heterocyclic pyrrol N—H forms an intermolecular water mediated hydrogen bond to one of the nitrogen atoms (N8) in the 6-membered pyrmidine heterocycle with a length of 2.78 (2) Å for the N—H···O and 2.86 (8) Å for the O—H···N contact. The water molecule connecting two molecules is disorderd over two positions (s.o.f. 0.48/0.52). The crystal packing is shows N—H···OH···N hydrogen bonds resulting in infinite chains parallel to the *a* axis.

### 2. Experimental

In an HPLC-vial, (3*R*,4*R*)-*N*,4-dimethyl-N-{7*H*-pyrrolo[2,3-*d*]pyrimidin-4- yl}piperidin-3-amine (50.0 mg, 204 µmol) and (oxetan-3-ylidene)acetonitrile (19.9 mg, 210 µmol) were dissolved in dry ethanol (400 µl) and the mixture stirred at 323 K during 48 h. The solvent was evaporated under reduced pressure and the product purified by column chromatography (SiO_2_, ethyl acetate + 2% methanol). The product was obtained as off-white solid (57.0 mg, 82%). Crystals of the title compound were obtained by slow evaporation of a solution in chloroform + 10% methanol at 298 K.

### 3. Refinement

Site occupation factors of the disordered water molecule were fixed assuming similar isotropic displacement parameters for alternative positions of the oxygen atom. Hydrogen atoms attached to carbons were placed at calculated positions with C—H = 0.95 Å (aromatic) or 0.99–1.00 Å (*sp*^3^ C-atom). All H atoms were refined with isotropic displacement parameters (set at 1.2–1.5 times of the *U*_eq_ of the parent atom). One of the H atoms of the disordered water molecule could be position to make a hydrogen bond to an N atom. The other one was positioneded with idealized geometric with respect to the first one. The absolute configuration was assigned according to the synthesis.

### Crystal data

**Table:**

C_18_H_24_N_6_O·H_2_O	*F*(000) = 768
*M**_r_* = 358.45	*D*_x_ = 1.324 Mg m^−^^3^
Orthorhombic, *P*2_1_2_1_2_1_	Mo *K*α radiation, λ = 0.71073 Å
Hall symbol: P 2ac 2ab	Cell parameters from 6886 reflections
*a* = 6.6088 (6) Å	θ = 2.5–27.8°
*b* = 10.1483 (8) Å	µ = 0.09 mm^−^^1^
*c* = 26.813 (2) Å	*T* = 193 K
*V* = 1798.3 (3) Å^3^	Plate, colourless
*Z* = 4	0.29 × 0.27 × 0.06 mm

### Data collection

**Table:**

Stoe IPDS 2T diffractometer	1716 reflections with *I* > 2σ(*I*)
Radiation source: sealed Tube	*R*_int_ = 0.079
Graphite monochromator	θ_max_ = 28.0°, θ_min_ = 3.2°
Detector resolution: 6.67 pixels mm^-1^	*h* = −7→8
rotation method scans	*k* = −11→13
6672 measured reflections	*l* = −29→35
4184 independent reflections	

### Refinement

**Table:**

Refinement on *F*^2^	Primary atom site location: structure-invariant direct methods
Least-squares matrix: full	Secondary atom site location: difference Fourier map
*R*[*F*^2^ > 2σ(*F*^2^)] = 0.062	Hydrogen site location: inferred from neighbouring sites
*wR*(*F*^2^) = 0.150	H-atom parameters constrained
*S* = 0.90	*w* = 1/[σ^2^(*F*_o_^2^) + (0.0523*P*)^2^] where *P* = (*F*_o_^2^ + 2*F*_c_^2^)/3
4184 reflections	(Δ/σ)_max_ < 0.001
246 parameters	Δρ_max_ = 0.19 e Å^−^^3^
0 restraints	Δρ_min_ = −0.22 e Å^−^^3^

### Special details

**Table:**

Geometry. All e.s.d.'s (except the e.s.d. in the dihedral angle between two l.s. planes) are estimated using the full covariance matrix. The cell e.s.d.'s are taken into account individually in the estimation of e.s.d.'s in distances, angles and torsion angles; correlations between e.s.d.'s in cell parameters are only used when they are defined by crystal symmetry. An approximate (isotropic) treatment of cell e.s.d.'s is used for estimating e.s.d.'s involving l.s. planes.
Refinement. Refinement of *F*^2^ against ALL reflections. The weighted *R*-factor *wR* and goodness of fit *S* are based on *F*^2^, conventional *R*-factors *R* are based on *F*, with *F* set to zero for negative *F*^2^. The threshold expression of *F*^2^ > σ(*F*^2^) is used only for calculating *R*-factors(gt) *etc*. and is not relevant to the choice of reflections for refinement. *R*-factors based on *F*^2^ are statistically about twice as large as those based on *F*, and *R*- factors based on ALL data will be even larger.

### Fractional atomic coordinates and isotropic or equivalent isotropic displacement parameters (Å^2^)

**Table:**

	*x*	*y*	*z*	*U*_iso_*/*U*_eq_	Occ. (<1)
N1	0.1280 (5)	0.1062 (4)	0.42472 (12)	0.0485 (10)	
H1	0.1160	0.1276	0.4564	0.058*	
C2	0.2919 (7)	0.0433 (4)	0.40332 (16)	0.0485 (12)	
H2	0.4106	0.0163	0.4205	0.058*	
C3	0.2563 (7)	0.0264 (4)	0.35393 (15)	0.0439 (11)	
H3	0.3446	−0.0139	0.3305	0.053*	
C4	0.0579 (7)	0.0814 (4)	0.34361 (15)	0.0423 (11)	
C5	−0.0680 (7)	0.1060 (4)	0.30217 (14)	0.0391 (10)	
N6	−0.2486 (6)	0.1667 (4)	0.30983 (12)	0.0449 (9)	
C7	−0.2941 (7)	0.2044 (4)	0.35573 (15)	0.0476 (11)	
H7	−0.4211	0.2473	0.3591	0.057*	
N8	−0.1909 (6)	0.1916 (4)	0.39726 (12)	0.0470 (9)	
C9	−0.0105 (7)	0.1291 (4)	0.38869 (14)	0.0413 (10)	
N10	−0.0216 (5)	0.0778 (3)	0.25423 (11)	0.0396 (8)	
C11	0.1748 (6)	0.0150 (4)	0.24320 (14)	0.0451 (11)	
H11A	0.1885	0.0029	0.2071	0.068*	
H11B	0.1815	−0.0709	0.2598	0.068*	
H11C	0.2847	0.0712	0.2554	0.068*	
C12	−0.1610 (7)	0.1075 (4)	0.21317 (14)	0.0415 (10)	
H12	−0.2699	0.1634	0.2281	0.050*	
C13	−0.0683 (7)	0.1906 (4)	0.17183 (13)	0.0419 (11)	
H13A	−0.1782	0.2339	0.1528	0.050*	
H13B	0.0160	0.2606	0.1870	0.050*	
N14	0.0562 (5)	0.1135 (3)	0.13743 (11)	0.0401 (9)	
C15	−0.0684 (7)	0.0153 (4)	0.11233 (14)	0.0437 (11)	
H15A	0.0130	−0.0318	0.0870	0.052*	
H15B	−0.1832	0.0588	0.0953	0.052*	
C16	−0.1472 (7)	−0.0818 (4)	0.15091 (15)	0.0474 (11)	
H16A	−0.0314	−0.1284	0.1663	0.057*	
H16B	−0.2331	−0.1483	0.1341	0.057*	
C17	−0.2698 (7)	−0.0137 (4)	0.19154 (14)	0.0424 (11)	
H17	−0.3970	0.0189	0.1755	0.051*	
C18	−0.3324 (7)	−0.1107 (5)	0.23178 (15)	0.0506 (11)	
H18A	−0.4324	−0.1724	0.2182	0.076*	
H18B	−0.3920	−0.0627	0.2599	0.076*	
H18C	−0.2135	−0.1598	0.2432	0.076*	
C19	0.1743 (7)	0.1946 (4)	0.10400 (14)	0.0421 (10)	
C20	0.3401 (7)	0.2731 (5)	0.12998 (15)	0.0528 (13)	
H20A	0.3255	0.3696	0.1259	0.063*	
H20B	0.3571	0.2497	0.1656	0.063*	
O21	0.4946 (5)	0.2159 (3)	0.09772 (13)	0.0672 (10)	
C22	0.3494 (7)	0.1203 (5)	0.07878 (17)	0.0539 (12)	
H22A	0.3701	0.0303	0.0920	0.065*	
H22B	0.3400	0.1190	0.0419	0.065*	
C23	0.0478 (7)	0.2774 (5)	0.06772 (15)	0.0472 (12)	
H23A	−0.0318	0.2179	0.0460	0.057*	
H23B	−0.0486	0.3322	0.0869	0.057*	
C24	0.1729 (8)	0.3629 (5)	0.03667 (16)	0.0484 (12)	
N25	0.2739 (7)	0.4295 (4)	0.01284 (15)	0.0658 (12)	
O1L	0.0901 (13)	0.1801 (9)	0.5242 (3)	0.077 (2)	0.48
H1L1	0.1783	0.2403	0.5036	0.115*	0.48
H1L2	0.1406	0.1662	0.5525	0.115*	0.48
O2L	0.1719 (14)	0.2748 (9)	0.5074 (2)	0.085 (2)	0.52
H2L1	0.0459	0.2619	0.5042	0.128*	0.52
H2L2	0.1751	0.3371	0.5283	0.128*	0.52

### Atomic displacement parameters (Å^2^)

**Table:**

	*U*^11^	*U*^22^	*U*^33^	*U*^12^	*U*^13^	*U*^23^
N1	0.062 (3)	0.052 (3)	0.0307 (18)	−0.009 (2)	−0.0069 (18)	0.0009 (18)
C2	0.048 (3)	0.051 (3)	0.047 (3)	0.001 (2)	0.002 (2)	−0.001 (2)
C3	0.049 (3)	0.044 (3)	0.038 (2)	−0.002 (2)	−0.001 (2)	0.002 (2)
C4	0.049 (3)	0.041 (3)	0.037 (2)	−0.006 (2)	−0.002 (2)	0.0023 (19)
C5	0.053 (3)	0.033 (2)	0.032 (2)	−0.003 (2)	0.0015 (19)	−0.0006 (19)
N6	0.046 (2)	0.052 (2)	0.0373 (19)	0.0060 (19)	0.0052 (17)	−0.0011 (17)
C7	0.056 (3)	0.050 (3)	0.037 (2)	0.002 (2)	0.005 (2)	−0.000 (2)
N8	0.059 (3)	0.048 (2)	0.0340 (18)	−0.004 (2)	0.0029 (19)	−0.0006 (16)
C9	0.052 (3)	0.039 (3)	0.032 (2)	−0.004 (2)	0.001 (2)	0.0042 (19)
N10	0.041 (2)	0.047 (2)	0.0304 (17)	0.0066 (18)	0.0005 (15)	−0.0002 (16)
C11	0.046 (3)	0.051 (3)	0.038 (2)	0.006 (2)	0.003 (2)	0.001 (2)
C12	0.044 (3)	0.043 (3)	0.037 (2)	0.005 (2)	−0.003 (2)	0.001 (2)
C13	0.048 (3)	0.047 (3)	0.031 (2)	0.008 (2)	−0.0015 (19)	−0.001 (2)
N14	0.050 (2)	0.037 (2)	0.0333 (17)	0.0038 (19)	−0.0005 (16)	−0.0040 (16)
C15	0.055 (3)	0.038 (3)	0.039 (2)	−0.000 (2)	−0.003 (2)	−0.005 (2)
C16	0.059 (3)	0.042 (3)	0.041 (2)	−0.001 (2)	−0.002 (2)	0.002 (2)
C17	0.046 (3)	0.042 (3)	0.040 (2)	0.002 (2)	−0.001 (2)	0.001 (2)
C18	0.053 (3)	0.055 (3)	0.044 (2)	0.001 (3)	−0.007 (2)	0.006 (2)
C19	0.044 (3)	0.047 (3)	0.035 (2)	−0.005 (2)	−0.002 (2)	0.003 (2)
C20	0.050 (3)	0.061 (3)	0.048 (3)	−0.004 (3)	−0.000 (2)	−0.002 (2)
O21	0.045 (2)	0.081 (3)	0.076 (2)	−0.002 (2)	0.0006 (18)	−0.005 (2)
C22	0.054 (3)	0.055 (3)	0.052 (3)	0.010 (3)	0.009 (2)	0.002 (2)
C23	0.051 (3)	0.051 (3)	0.040 (2)	−0.001 (2)	−0.001 (2)	0.012 (2)
C24	0.060 (3)	0.048 (3)	0.037 (2)	0.013 (3)	0.002 (2)	−0.004 (2)
N25	0.084 (3)	0.059 (3)	0.054 (2)	−0.003 (2)	0.014 (2)	0.006 (2)
O1L	0.113 (7)	0.072 (6)	0.045 (4)	0.005 (5)	−0.013 (4)	0.001 (4)
O2L	0.130 (7)	0.078 (6)	0.049 (4)	0.010 (5)	−0.020 (5)	−0.024 (4)

### Geometric parameters (Å, º)

**Table:**

N1—C9	1.351 (5)	C15—H15A	0.9900
N1—C2	1.381 (5)	C15—H15B	0.9900
N1—H1	0.8800	C16—C17	1.524 (6)
C2—C3	1.356 (6)	C16—H16A	0.9900
C2—H2	0.9500	C16—H16B	0.9900
C3—C4	1.452 (6)	C17—C18	1.519 (6)
C3—H3	0.9500	C17—H17	1.0000
C4—C9	1.378 (5)	C18—H18A	0.9800
C4—C5	1.410 (6)	C18—H18B	0.9800
C5—N10	1.352 (5)	C18—H18C	0.9800
C5—N6	1.359 (5)	C19—C20	1.523 (6)
N6—C7	1.323 (5)	C19—C23	1.534 (6)
C7—N8	1.312 (5)	C19—C22	1.538 (6)
C7—H7	0.9500	C20—O21	1.458 (5)
N8—C9	1.369 (6)	C20—H20A	0.9900
N10—C12	1.467 (5)	C20—H20B	0.9900
N10—C11	1.476 (5)	O21—C22	1.456 (6)
C11—H11A	0.9800	C22—H22A	0.9900
C11—H11B	0.9800	C22—H22B	0.9900
C11—H11C	0.9800	C23—C24	1.459 (7)
C12—C13	1.521 (6)	C23—H23A	0.9900
C12—C17	1.538 (6)	C23—H23B	0.9900
C12—H12	1.0000	C24—N25	1.145 (6)
C13—N14	1.463 (5)	O1L—H1L1	1.0100
C13—H13A	0.9900	O1L—H1L2	0.8390
C13—H13B	0.9900	O1L—H2L1	1.0319
N14—C19	1.445 (5)	O2L—H1L1	0.3669
N14—C15	1.458 (5)	O2L—H2L1	0.8478
C15—C16	1.520 (6)	O2L—H2L2	0.8441
			
C9—N1—C2	108.3 (3)	H15A—C15—H15B	108.3
C9—N1—H1	125.8	C15—C16—C17	112.0 (4)
C2—N1—H1	125.8	C15—C16—H16A	109.2
C3—C2—N1	109.2 (4)	C17—C16—H16A	109.2
C3—C2—H2	125.4	C15—C16—H16B	109.2
N1—C2—H2	125.4	C17—C16—H16B	109.2
C2—C3—C4	107.1 (4)	H16A—C16—H16B	107.9
C2—C3—H3	126.4	C18—C17—C16	111.0 (4)
C4—C3—H3	126.4	C18—C17—C12	112.2 (3)
C9—C4—C5	115.8 (4)	C16—C17—C12	112.6 (4)
C9—C4—C3	105.3 (4)	C18—C17—H17	106.9
C5—C4—C3	138.7 (4)	C16—C17—H17	106.9
N10—C5—N6	116.0 (4)	C12—C17—H17	106.9
N10—C5—C4	125.3 (4)	C17—C18—H18A	109.5
N6—C5—C4	118.6 (4)	C17—C18—H18B	109.5
C7—N6—C5	118.1 (4)	H18A—C18—H18B	109.5
N8—C7—N6	130.0 (4)	C17—C18—H18C	109.5
N8—C7—H7	115.0	H18A—C18—H18C	109.5
N6—C7—H7	115.0	H18B—C18—H18C	109.5
C7—N8—C9	110.8 (4)	N14—C19—C20	113.7 (3)
N1—C9—N8	123.3 (4)	N14—C19—C23	114.2 (4)
N1—C9—C4	110.1 (4)	C20—C19—C23	113.3 (4)
N8—C9—C4	126.5 (4)	N14—C19—C22	113.6 (4)
C5—N10—C12	121.8 (3)	C20—C19—C22	85.2 (3)
C5—N10—C11	118.8 (3)	C23—C19—C22	113.6 (3)
C12—N10—C11	119.4 (3)	O21—C20—C19	91.4 (3)
N10—C11—H11A	109.5	O21—C20—H20A	113.4
N10—C11—H11B	109.5	C19—C20—H20A	113.4
H11A—C11—H11B	109.5	O21—C20—H20B	113.4
N10—C11—H11C	109.5	C19—C20—H20B	113.4
H11A—C11—H11C	109.5	H20A—C20—H20B	110.7
H11B—C11—H11C	109.5	C22—O21—C20	90.6 (3)
N10—C12—C13	114.1 (3)	O21—C22—C19	90.9 (3)
N10—C12—C17	114.3 (3)	O21—C22—H22A	113.5
C13—C12—C17	110.9 (3)	C19—C22—H22A	113.5
N10—C12—H12	105.5	O21—C22—H22B	113.5
C13—C12—H12	105.5	C19—C22—H22B	113.5
C17—C12—H12	105.5	H22A—C22—H22B	110.8
N14—C13—C12	112.9 (4)	C24—C23—C19	112.2 (4)
N14—C13—H13A	109.0	C24—C23—H23A	109.2
C12—C13—H13A	109.0	C19—C23—H23A	109.2
N14—C13—H13B	109.0	C24—C23—H23B	109.2
C12—C13—H13B	109.0	C19—C23—H23B	109.2
H13A—C13—H13B	107.8	H23A—C23—H23B	107.9
C19—N14—C15	114.1 (3)	N25—C24—C23	178.8 (5)
C19—N14—C13	113.0 (3)	H1L1—O1L—H1L2	111.5
C15—N14—C13	109.8 (3)	H1L1—O1L—H2L1	52.4
N14—C15—C16	108.8 (3)	H1L2—O1L—H2L1	135.9
N14—C15—H15A	109.9	H1L1—O2L—H2L1	86.4
C16—C15—H15A	109.9	H1L1—O2L—H2L2	153.9
N14—C15—H15B	109.9	H2L1—O2L—H2L2	102.0
C16—C15—H15B	109.9		
			
C9—N1—C2—C3	−0.2 (5)	C12—C13—N14—C19	−169.0 (3)
N1—C2—C3—C4	−0.2 (5)	C12—C13—N14—C15	62.3 (4)
C2—C3—C4—C9	0.5 (5)	C19—N14—C15—C16	167.6 (4)
C2—C3—C4—C5	174.8 (5)	C13—N14—C15—C16	−64.4 (4)
C9—C4—C5—N10	174.1 (4)	N14—C15—C16—C17	58.3 (5)
C3—C4—C5—N10	0.2 (8)	C15—C16—C17—C18	−175.4 (4)
C9—C4—C5—N6	−3.7 (6)	C15—C16—C17—C12	−48.6 (5)
C3—C4—C5—N6	−177.5 (5)	N10—C12—C17—C18	39.5 (5)
N10—C5—N6—C7	−175.4 (4)	C13—C12—C17—C18	170.1 (4)
C4—C5—N6—C7	2.6 (6)	N10—C12—C17—C16	−86.7 (4)
C5—N6—C7—N8	−0.7 (7)	C13—C12—C17—C16	44.0 (5)
N6—C7—N8—C9	0.1 (7)	C15—N14—C19—C20	−166.0 (4)
C2—N1—C9—N8	−179.3 (4)	C13—N14—C19—C20	67.7 (5)
C2—N1—C9—C4	0.5 (5)	C15—N14—C19—C23	61.8 (5)
C7—N8—C9—N1	178.2 (4)	C13—N14—C19—C23	−64.6 (5)
C7—N8—C9—C4	−1.5 (6)	C15—N14—C19—C22	−70.7 (5)
C5—C4—C9—N1	−176.4 (4)	C13—N14—C19—C22	162.9 (3)
C3—C4—C9—N1	−0.7 (5)	N14—C19—C20—O21	123.8 (4)
C5—C4—C9—N8	3.3 (6)	C23—C19—C20—O21	−103.5 (4)
C3—C4—C9—N8	179.1 (4)	C22—C19—C20—O21	10.2 (3)
N6—C5—N10—C12	−1.8 (6)	C19—C20—O21—C22	−10.7 (3)
C4—C5—N10—C12	−179.6 (4)	C20—O21—C22—C19	10.6 (3)
N6—C5—N10—C11	178.5 (4)	N14—C19—C22—O21	−124.0 (4)
C4—C5—N10—C11	0.6 (6)	C20—C19—C22—O21	−10.2 (3)
C5—N10—C12—C13	125.6 (4)	C23—C19—C22—O21	103.2 (4)
C11—N10—C12—C13	−54.6 (5)	N14—C19—C23—C24	176.8 (4)
C5—N10—C12—C17	−105.3 (4)	C20—C19—C23—C24	44.4 (5)
C11—N10—C12—C17	74.5 (5)	C22—C19—C23—C24	−50.7 (5)
N10—C12—C13—N14	79.8 (4)	C19—C23—C24—N25	−12 (26)
C17—C12—C13—N14	−51.0 (5)		

### Hydrogen-bond geometry (Å, º)

**Table:**

*D*—H···*A*	*D*—H	H···*A*	*D*···*A*	*D*—H···*A*
N1—H1···O1*L*	0.88	1.90	2.783 (8)	178
N1—H1···O2*L*	0.88	2.06	2.816 (7)	144
O1*L*—H1*L*2···N8^i^	0.84	2.27	2.868 (8)	129
O2*L*—H2*L*2···N8^i^	0.84	2.20	2.733 (7)	121
O2*L*—H2*L*2···N25^ii^	0.84	2.43	3.026 (10)	129

Symmetry codes: (i) *x*+1/2, −*y*+1/2, −*z*+1; (ii) −*x*+1/2, −*y*+1, *z*+1/2.
